# Multiple Fitness Benefits of Polyandry in a Cephalopod

**DOI:** 10.1371/journal.pone.0037074

**Published:** 2012-05-16

**Authors:** Zoe E. Squires, Bob B. M. Wong, Mark D. Norman, Devi Stuart-Fox

**Affiliations:** 1 Zoology Department, University of Melbourne, Melbourne, Victoria, Australia; 2 School of Biological Sciences, Monash University, Melbourne, Victoria, Australia; 3 Sciences Department, Museum Victoria, Melbourne, Victoria, Australia; University of Arizona, United States of America

## Abstract

**Background:**

Sex differences in reproductive investment play a crucial role in sexual conflict. One intriguing aspect of sexual conflict is the evolution of female multiple mating (polyandry), particularly in systems where females receive no obvious direct benefits from males, and where mating is highly costly. Here, theory predicts that polyandrous females can increase their reproductive success by taking advantage of the genetic benefits of mating with multiple males. Cephalopods provide a model system for addressing this question, as all species mate multiply. Here we examine differences in reproductive success between monandrous, multiply mated (to the same male) and polyandrous female dumpling squid (*Euprymna tasmanica*).

**Methodology/Principal Findings:**

We mated females in the laboratory with two different males (polyandrous; controlling for mating order), or with a single male (monandrous). To control for mating frequency, we mated monandrous females either once (monandrous 1), or with the same male twice (monandrous 2), and measured reproductive success for each of the three treatments (polyandrous, monandrous 1, monandrous 2). Females mated to two different males produced eggs faster and had larger hatchlings relative to egg mass than females mated once with a single male.

**Conclusions/Significance:**

The benefits of polyandry demonstrated here are the first, to our knowledge, in any cephalopod. These benefits may outweigh the significant costs associated with mating and help to explain how multiple mating has evolved (or is maintained) in this group.

## Introduction

The adaptive significance of polyandry (where females have two or more mates) is currently under considerable debate [1], [2], especially in systems where females receive no obvious direct benefits from mating [3]–[5]. It is well established that mating can be extremely costly [6]–[8] and that differences in reproductive investment between the sexes can often lead to sexual conflict over mating frequency [9]. However, the ubiquity of polyandry across the animal kingdom suggests that, despite such costs, mating multiply can also confer important fitness benefits to females [2]. Such benefits include increasing the rate of reproduction [10], [11], producing larger or fitter offspring [12]–[15], increasing hatching success [2], [16], and/or increasing overall fecundity [17], [18]. Maternal investment in reproduction also has important consequences for reproductive output [15], [19], [20]. This, too, can be influenced by mating frequency [19]. Determining whether there are fitness benefits of polyandry and distinguishing which components of reproduction are influenced, is important for understanding how mating systems evolve or are maintained [1].

When determining the fitness benefits of polyandry, it is important to be able to disentangle the effects of mating polyandrously from the effects of multiple matings *per se*. In some systems, the benefits of polyandry may be driven solely by the quantity of sperm received (e.g. by replenishing sperm stores). In addition, recent studies have highlighted the importance of accessory seminal proteins that may function to increase the reproductive fitness of multiply mating females [21] by providing nutrients [18], [22] or by manipulating female reproductive output [10]. Alternatively, the diversity or quality of sperm received may be the important driver of any fitness benefits to polyandry. By mating with multiple males, females may take advantage of post-copulatory mechanisms, such as sperm competition and cryptic female choice of sperm, that bias fertilisation towards males with intrinsically good genes (‘good genes’ hypothesis; for reviews see [1], [23]), compatible genes (‘genetic compatibility’ hypothesis; for reviews see [4], [24]) or both. The effects of these drivers need not be mutually exclusive. For example, in the Parthenium beetle (*Zygogramma bicolorata*), polyandrous females have higher fecundity and their offspring develop faster, benefits that appear to be driven by an increased supply of nutrients transferred in the ejaculate (direct benefits) and genetic (indirect) benefits respectively [25].

Cephalopod reproductive behaviour and morphology suggests the potential for strong sexual selection and sexual conflict. All species studied to date mate multiply [26]–[29], with an average of 2–4 males contributing to clutches in those species where it has been measured [28], [30]–[32]. Male cephalopods typically transfer numerous, large spermatophores using a modified arm (the hectocotylus), which in some species have scoops, plates or suckers that may be able to break open spermatophores from their competitors [26], [33], [34]. Females can also store sperm in numbers and for time frames much greater than is necessary to fertilise all their eggs [33]. Females of many species have deeply envaginated and muscular sperm storage organs, which have led to the hypothesis that females have control over which male's sperm they use to fertilise their eggs [26]. Copulations can be lengthy and, in some species, females can incur tentacle scars and cuts from spermatophore insertion [35]. Yet, despite ubiquitous polyandry and strong potential for sexual conflict over mating frequency, to our knowledge, the fitness benefits of polyandry in cephalopods has not been reported.

**Figure 1:**
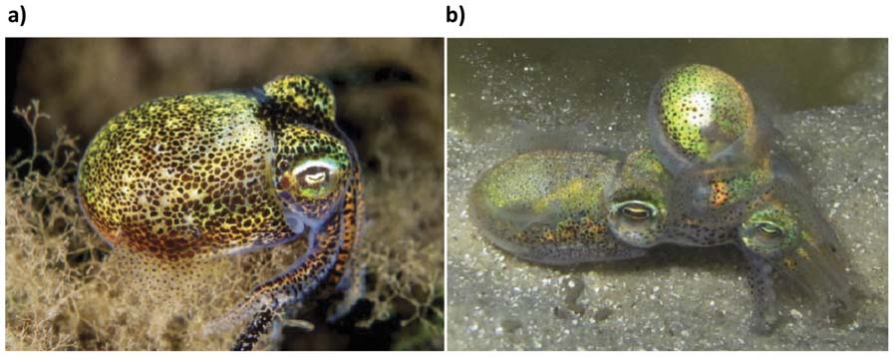
Photos of *Euprymna tasmanica* in the field. a) individual squid (photo: Mark Norman) b) mating pair (male left, photo: Zoe Squires).

The aim of the current study was to experimentally investigate the benefits of polyandry in *Euprymna tasmanica* (dumpling squid, Figure 1a)), a small, semi-solitary, nocturnal and benthic species native to southern Australia. *Euprymna tasmanica* is a model species with which to assess the benefits of polyandry and multiple mating because both sexes mate multiply, copulation is lengthy (mean = 92±4.3 mins) and they are highly amenable to captivity. During mating (Figure 1b), males transfer numerous large spermatophores (approximately 20 per mating) and females are capable of sperm storage [36]. Females lay multiple clutches of opaque orange eggs in clusters (mean = 50, range = 6–109). In early life stages, cephalopods shift from a yolk-utilisation phase to active predation. This is a particularly critical period affecting survival [37]–[39]. As such, egg size, yolk reserves and size at hatching are important life history traits. Here we report an experiment testing the effect of both mating frequency (mating with a single male or mating with the same male twice) and polyandry (mating with two different males) on a number of female reproductive components including overall fecundity, rate of reproduction, hatching success and hatchling size. Importantly, our experiment controlled for both the number of mates and the order in which they were encountered.

## Results

### Reproductive Success

**Figure 2:**
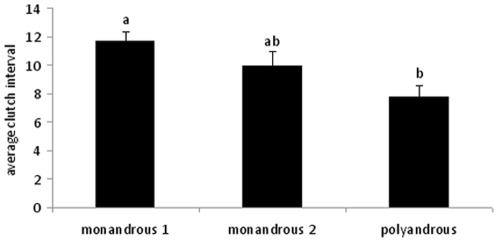
The average clutch interval (days) between monandrous 1, monandrous 2 and polyandrous females. Different letters show significantly different groups.

Mating treatment had a significant effect on mean reproductive success measures (MANOVA: Wilks' Lambda = 0.395, F_14, 48_ = 2.03, P = 0.036). One-way ANOVAs revealed a significant effect of treatment on inter-clutch interval (the average number of days between each clutch) (F_2, 31_ = 7.74, P = 0.002; Figure 2). Polyandrous females produced clutches faster than monandrous 1 females (Tukey's post-hoc test: t = −3. 042, P = 0.013). Monandrous 2 females produced clutches at an intermediate rate, which did not differ significantly from that of either polyandrous females (t = −1.413, P = 0.35) or monandrous 1 females (t = −1.684, P = 0.23).

**Figure 3:**
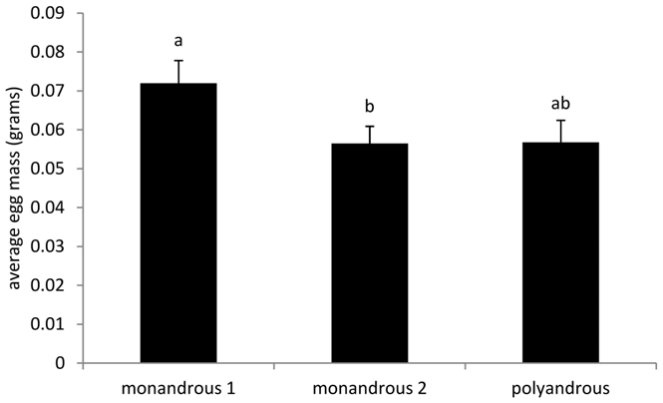
The average egg mass (grams) between monandrous 1, monandrous 2 and polyandrous females. Different letters show significantly different groups.

There was a significant effect of treatment on the average egg mass produced (F_2, 31_ = 5.429, P = 0.009; Figure 3), with females that had mated only once, producing significantly larger eggs than monandrous 2 females (t = −2.838, P = 0.021). Additionally, there was no statistical difference among treatments in the latency to lay the first clutch (F_2, 36_ = 2.35, P = 0.11; Table 1).

**Table 1:** Reproductive success of monandrous and polyandrous female *E. tasmanica*.

Variable	Mating treatment (means and standards errors)			ANOVA
	Monandrous 1 (n = 18)	Monandrous 2 (n = 13)	Polyandrous (n = 14)	
number of clutches	5.17±0.78	4.69±0.76	4.79±0.98	F_2,42_ = 0.01, p = 0.98
total number of eggs	199.73±31.76	214.39±34.20	182±35.64	F_2,41_ = 0.48, p = 0.62
lay latency (days)	19±3.16	11±2.05	13±2.77	F_2,36_ = 2.35, p = 0.11
egg mass (grams)	0.08±0.01	0.06±0.004	0.06±0.01	F_2,31_ = 5.43, p = 0.01
inter-clutch interval (days)	12.27±0.52	9.92±0.91	8.40±0.90	F_2,31_ = 7.74, p = 0.002
egg development time (days)	39.44±1.49	39.53±1.13	35.69±0.88	F_2,34_ = 2.23, p = 0.12
proportion hatched	0.63±0.06	0.65±0.07	0.62±0.04	F_2,34_ = 0.14, p = 0.87
hatchling mass (grams)	0.0141±0.0005	0.0136±0.0005	0.0154±0.0003	F_2,25_ = 1.94, p = 0.17
hatchling mass (grams)/egg mass (grams)	0.218±0.025	0.226±0.0113	0.252±0.0156	F_2,25_ = 3.80, p = 0.04

**Figure 4:**
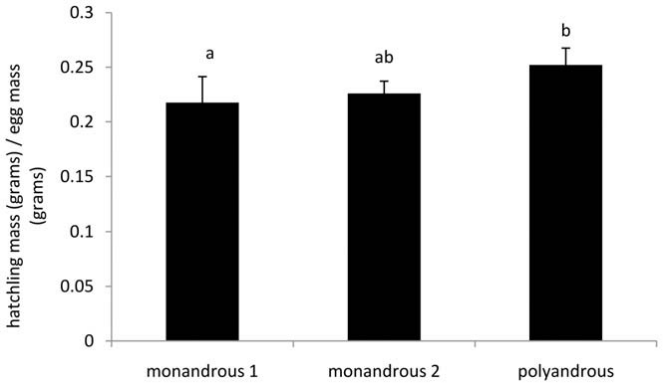
Hatchling mass (grams) as a function of egg mass (grams) for monandrous 1, monandrous 2 and polyandrous females. Different letters show significantly different groups.

Even though once-mated females produced the largest eggs, and egg mass and hatchling mass were significantly correlated (F_1, 130_ = 25.43, P<0.001, R^2^ = 0.16), there was no overall effect of treatment on hatchling mass (F_25, 102_ = 1.678, P = 0.21). We therefore investigated the relationship between egg mass and hatchling mass further and found that mating treatment significantly influenced hatchling mass relative to egg mass (F_1, 103_ = 3.532, P = 0.044; Figure 4). Tukey's tests revealed that polyandrous females produced significantly larger hatchlings relative to egg mass than monandrous 1 females (t = 2.361, P<0.026). There was a trend for monandrous 2 females to also produce larger hatchlings relative to egg mass than monandrous 1 females (t = 2.018, P = 0.052) and no difference between polyandrous and monandrous 2 females (t = −0.417, P = 0.68). Furthermore, egg size significantly predicted hatchling size for monandrous 1 (R^2^ = 0.18, p<0.001, F_1, 56_ = 13.64) and monandrous 2 (R^2^ = 0.074, p = 0.048, F_1, 39_ = 4.173) females but not polyandrous females (R^2^ = 0.0271, p = 0.179, F_1, 31_ = 1.89).

There was no effect of treatment on the other reproductive fitness variables (Table 1) and no difference in the number of females from each treatment that failed to reproduce (monandrous 1 = 16.67%, monandrous 2 = 7.69%, polyandrous = 14.29%). Finally, there was no significant effect of mating treatment on the variance of reproductive variables (MANOVA: Wilks' Lambda = 0.77, F_10, 38_ = 0.53, P = 0.86; Table 2).

**Table 2:** Variance in reproductive success measures of monandrous and polyandrous female *E. tasmanica*.

Variable	Mating treatment (means of the standard deviation and se of the standard deviation)			ANOVA
	Monandrous 1 (n = 18)	Monandrous 2 (n = 13)	Polyandrous (n = 14)	
egg mass (grams)	0.0199±0.0024	0.0179±0.0027	0.0147±0.0026	F_2,23_ = 0.66, p = 0.53
inter-clutch interval	45.16±3.63	17.77±6.81	21.23±5.17	F_2,23_ = 2.83, p 0.08
egg development time (days)	18.18±4.71	25.16±9.57	18.41±6.39	F_2,23_ = 0.01, p = 0.99
proportion hatched	0.2294±0.0553	0.1429±0.0640	0.1516±0.0439	F_2,23_ = 0.43, p = 0.66
hatchlings mass (grams)	0.0024±0.0004	0.0022±0.0001	0.0021±0.0002	F_2,12_ = 0.50, p = 0.62
hatchling mass (grams)/egg mass (grams)	0.0409±0.0075	0.0667±0.0182	0.0471±0.0110	F_2,19_ = 0.33, p = 0.72

## Discussion

Polyandry provides female *E. tasmanica* with potential fitness benefits that may outweigh the costs associated with mating. To our knowledge, whilst polyandry, multiple paternity and other aspects of sexual selection have received some attention in cephalopods [32], [34], these are the first potential benefits of polyandry demonstrated for this group. Firstly, polyandrous *E. tasmanica* females produce eggs at a faster rate than their monandrous counterparts which may significantly increase fecundity [40]. Furthermore, compared to once-mated females, polyandrous females produced hatchlings that were larger relative to egg mass. Hatchling size commonly determines hatchling survival in a range of species [41] because larger hatchlings have better prey capture abilities and can swim faster to avoid predation [42]. While polyandrous females did not produce larger hatchlings, they needed to invest less per egg to produce similarly sized hatchlings compared with monandrous females. Lower maternal investment per hatchling, combined with producing eggs at a faster rate, suggests that polyandrous females have the potential to produce more eggs over their reproductive lifetime and, therefore, probably have a higher reproductive fitness than monandrous females.

Polyandrous *E. tasmanica* produced eggs faster, representing a significant potential benefit of polyandry in this species. In our experiment, where reproductive longevity may be artificially extended due to the benign conditions afforded by captivity, there was no difference in the total number of eggs produced by females subjected to the different treatments. In the wild, however, where reproductive bouts may be cut short by predation, even a very small increase in the rate of reproduction can lead to a large increase in fecundity [40].

An increase in reproductive rate has previously been reported in a range of animals after polyandrous mating (guppies: 11; fruit flies: 10). In some cases, this has been attributed to manipulative substances transferred within seminal fluid [10], [22], [43]. The amount of sperm or manipulative accessory substances received could explain the observed pattern in reproductive rate among treatments. Specifically, the intermediate reproductive rate of females mated with the same male twice could be a potential consequence of receiving an intermediate quantity of sperm and accessory substances if males mated with a familiar female strategically allocate less sperm in the second mating [44].

Another explanation for increased reproductive rate in polyandrous females is that females may perceive that they are in an advantageous environment with multiple males, and invest more resources to reproduction. Alternatively, males may vary in the quality of their accessory seminal proteins such that the sperm of some males may be better able to manipulate female reproductive rate. Consequently, polyandry may allow females to receive more competitive (and effective) manipulating proteomes. Additionally, in some species, accessory seminal fluid contains nutrients [43] which females can use to increase their reproductive output. Considering the large size and number of spermatophores transferred in one mating in *E. tasmanica*, and the extensive surface area of the female sperm storage organ, it could well be that nutrients are contained in the sperm and females could absorb them through their reproductive tract. Indeed, substances that could be nutritious for the female (glucose, galactose, mannose, methyl pentose and amino sugar) have been found in spermatophoric plasma of other cephalopod species [45]. It appears likely that a combination of mechanisms is responsible for the observed increase in reproductive rate, particularly given the intermediate rate of females mated twice with the same male.

Egg size is traditionally thought to be driven by differential maternal investment – the bigger the egg the larger the reproductive investment [46] and the higher the quality of the egg [47], [48]. Previous studies have found that *E. tasmanica* females fed a low ration diet produced smaller eggs with lower lipid content than females fed a high ration diet [38]. Additionally, females fed on a higher ration diet also produced larger hatchlings independent of female size [38]. This indicates that egg size is a good proxy for maternal investment or egg quality in this species. Our data suggest that once-mated females may be investing more per egg compared with multiply mated females. If genetic benefits are important, females may compensate for mating with only one male (that may have bad or incompatible genes) and invest more in these clutches. These data suggest that mating once is a suboptimal mating strategy in this species because, despite producing larger eggs and an overall correlation between egg mass and hatchling mass, once-mated females did not produce larger hatchlings. In contrast to our data, previous studies have found that polyandrous females (sea slugs: [15],[14]; salmon:[49], [13], [12]; crickets: [50]; seed beetles: [51]) produced larger eggs than their monandrous counterparts. However, in each of these cases, these females also produced larger hatchlings. The mismatch between egg size and hatchling size in *E. tasmanica* may represent an important trade off between maternal investment and reproductive output, and warrants further investigation.

If egg mass reflects maternal investment, our data suggest that polyandrous *E. tasmanica* females do not need to invest as much as once-mated females to produce similarly sized hatchlings. A number of factors may be driving this effect. Mating multiply encourages sperm competition, whereby the competitive performance of a male's sperm may reflect the genetic quality of the father (‘good genes hypothesis’) and, as a result, produce higher quality offspring. Evidence suggests that the ability of an embryo to transform yolk energy into somatic growth may be genetically determined. For example, polyandry in Arctic charr (*Salvelinus alpines*) increases selection on genetically superior sperm which produces offspring that have better quality yolk reserves [12]. In a study investigating the effects of maternal hormones (cortisol and testosterone) on larval development in coral reef fish (*Pomacentrus amboinensis*) [52], a slight increase in testosterone levels in the egg increased yolk utilisation rates in embryos. It is therefore conceivable that hormones, either within the yolk or transferred by the male, alter the efficiency with which *E. tasmanica* embryos develop within the egg. As there was no difference in development time among treatments, it is possible that more efficient metabolism of yolk reserves in squid hatchlings allows offspring of polyandrous females to grow larger on fewer reserves and hatch at the same time as hatchlings from once-mated females. By mating multiply, females may be facilitating sperm competition that intensifies selection on genetically superior sperm and potentially producing offspring with superior yolk reserves, as has been found in Arctic charr [12]. Although the pattern was stronger for polyandrous females, there was a trend for females mated to the same male twice to also have larger hatchlings relative to egg size. This suggests that the amount of sperm, and not simply the diversity of sperm, may also play an important role in mediating the effect of multiple mating on relative hatchling size. This result may offer further support for the hypothesis of nutrient transfer from spermatophores. Alternatively, it may indicate that hormones important in affecting yolk utilisation in embryos (discussed above) may be transferred within the spermatophore. If this is the case, both the quantity and quality of sperm transferred is important.

One prediction of genetic benefits of polyandry hypotheses is that polyandry reduces the variance in reproductive output. For example, polyandry in the grey foam nesting tree frog (*Chiromantis xerampelina*), reduces the variance in offspring survival [53] therefore increasing female fecundity on average. We did not find any effect of mating treatment on the variance of any reproductive variables measured. Benefits of this kind may only be apparent under stressful conditions or in spatially or temporally heterogeneous environments, in contrast to laboratory conditions. The fact that we also found no difference in the proportion of eggs that hatched among treatments potentially suggests that any genetic benefits from polyandry are not influencing fertilisation success or ‘successful’ development within the egg in this species.

Fitness benefits gained by polyandrous *E. tasmanica* may be the result of a combination of indirect genetic benefits (receiving ‘good genes’) but also potentially direct benefits, such as nutrients transferred in the seminal fluid. To test these hypotheses, further experiments would be needed to track whether substances within spermatophores are absorbed by the female, or whether males that do better in sperm competition, also sire fitter sons. However, regardless of the mechanisms behind these fitness benefits, our data suggest that mating polyandrously (and to a lesser extent mating multiply) in *E. tasmanica*, provides females with potential fitness benefits, and, in so doing, offer important insights into how this mating strategy is selected or maintained.

## Materials and Methods

### Ethics statement

This study was carried out according to the University of Melbourne's animal ethics committee (ID: 0810874.2) and animals were collected under a Fisheries Victoria collecting permit (RP962).

### Squid collection and housing

We collected *E. tasmanica* using SCUBA from Port Phillip Bay (38°10.81′S, 144°44.60′E) in south-eastern Australia, between January 2009 and March 2010. Upon capture, squid were transported to the Victorian Marine Science Consortium facilities where they were housed in individual aquaria (length×width×depth = 24×24×24 cm, volume = 13.8 litres) containing a layer of sand substrate and a length of PVC pipe (6.5 cm long, 5.5 cm diameter) in which a female could lay eggs. Tanks were illuminated from above with aquarium lights on a 12 h day: 12 h night cycle and supplied with a continuous through-flow of sea water. This was pumped directly from Port Phillip Bay (temperature range = 13–20°C). Squid were fed live *Palaemon* sp. shrimp *ad libitum*, and were checked for eggs every second day. In some cephalopod species, including *E. tasmanica*, males mate with sub-adult females, which can store sperm. For this reason, it is exceedingly difficult to collect virgins from the wild. Our pilot studies showed that females collected from the wild generally laid viable eggs, within a median of 13 days of being in captivity (n = 11, range = 4–17 days). Therefore, we conservatively used females that had not laid any eggs by day 28 for mating trials. Subsequent experiments did show, however, that some females can occasionally lay viable clutches up to 82 days (n = 66 females, median lay latency = 15 days, range 0–82 days) after being brought into captivity. However, the potential for sperm storage and mating history to influence reproductive output will affect all treatments similarly. Our results are therefore likely to be robust. Squid were removed from aquaria, blotted, weighed and randomly assigned to treatments. There was no difference in water temperatures (mean = 17.64±0.34°C) among treatments (F_2, 42_ = 0.075, p = 0.93) and no difference in female mass (mean = 6.59±0.38 g) among treatments (F_2, 42_ = 0.1646, p = 0.85). Nevertheless, we included temperature and female mass as random effects in our models (see statistical analysis).

### Experimental Design

Each male was placed separately into a mating tank measuring 10×10×11.5 cm (volume = 1.5 litres) and left for a 10 min acclimation period before a female was introduced. If mating did not begin within 30 min, the female was gently disturbed from the bottom of the tank, which allowed the male to initiate mating. In most cases mating was successful; however in some cases (N = 16) mating did not commence for unknown reasons, and a different pair was then chosen. Females were randomly assigned to one of three treatments: lab mated once; lab mated twice, same male; or lab mated twice, different males. For brevity, we term females mated once in the lab as monandrous 1 (n = 18); females mated twice with the same males as monandrous 2 (n = 13) and females mated twice with different males as polyandrous (n = 14). This design controls for both mate history (females encountered one male that had already mated once and one male that had not yet mated) and mate order (the order the female encountered once mated or not yet mated males was randomised). As mating lasts up to two hours and has been shown to reduce swimming endurance in this species (Franklin, Squires and Stuart-Fox, unpublished data), squid were reluctant to mate more than twice in the lab, with survival substantially decreasing after the third mating. Thus, the twice-mated treatments are likely to reflect biologically relevant levels of multiple mating in this species [28], [30], [31]. A total of 45 males were used, 13 for the monandrous 2 treatment and the rest allocated to monandrous 1 females (only as first maters) and polyandrous females (second maters were reused from monandrous 1 treatment) or paired between two polyandrous females (one as a first mater for one female and a second mater for the other). In both the multiple mating treatments (monandrous 2 and polyandrous) matings occurred with one day break in between to minimise stress and avoid complete sperm depletion in the male. Males had been kept in the lab for a minimum of eight days prior to experiments to ensure that there was no recent depletion of spermatophores.

Following mating, squid were returned to their holding tanks and females left to lay eggs until senescence. Once this occurred, we removed egg clutches and blotted them to remove excess water before weighing the clutches and recording the number of eggs in each clutch. Each clutch was then placed individually into a constant temperature (19°C) holding tank and left to develop. Clutches were inspected every second day for hatchlings to calculate their developmental time (i.e. time to hatching). Any resulting hatchlings were counted and a subset of hatchlings (from 28 randomly selected females across the three treatments) were anaesthetised, blotted to remove excess water and weighed. Egg clutches and hatchlings were weighed to 0.0001 gram accuracy. In total, we measured nine reproductive variables: number of clutches, total number of eggs, latency to lay the first clutch (i.e. lay latency in days), mean egg mass (clutch mass/number of eggs), inter-clutch interval (days), egg development time (days), the proportion of eggs that hatched (number hatched/egg number), total number of hatchlings, and hatchling mass (grams).

### Statistical Analysis

Statistical analyses were performed in R (R Development Core Team, 2010). All data were checked for normality and homogeneity of variance, and all variables met these assumptions. To test whether mating treatment had any effect on reproductive success we conducted a MANOVA on the following seven reproductive variables: number of clutches, total number of eggs, lay latency, egg mass, inter-clutch interval, egg development time and the proportion hatched.

We did not include total number of hatchlings in the MANOVA as it is a function of two other included variables (total number of eggs and proportion hatched). We also did not include hatchling mass because we did not have a complete dataset for this variable. Instead, to see if mating treatment affected hatchling mass, we ran a linear mixed effects model, with clutch number nested within female ID to account for non-independence of multiple clutches per female. An important benefit of polyandry can be to reduce the variance in reproductive output [53], [54]. To test whether this was the case in dumpling squid we also conducted a MANOVA on the standard deviation per female for the following 4 reproductive variables; egg mass, inter-clutch interval, development time and proportion hatched. We only included those variables measured for individual clutches (i.e. with more than two values per female), and therefore did not include number of clutches, total number of eggs, lay latency and total number of hatchlings. Where MANOVAs were significant, we ran one-way ANOVAs to further investigate the effect of mating treatment on each measure of reproductive success. We included temperature and female mass as covariates in initial ANOVA models but excluded them from final models if they had no significant effect. Temperature and female mass were therefore included for egg mass data, and temperature included for average clutch interval data.
